# Draft Genome Sequence of *Lactobacillus brevis* Strain 3M004, a Probiotic with Potential Quorum-Sensing Regulation

**DOI:** 10.1128/genomeA.00675-17

**Published:** 2017-09-07

**Authors:** Qichuang Li, Yonglong Pan, Linxian Ding, Huachang Hong, Shuxia Yan, Binbin Wu, Yan Liang

**Affiliations:** a Zhejiang Normal University, Jinhua, People’s Republic of China; b Shenzhen Institutes of Advanced Technology, Chinese Academy of Sciences, People’s Republic of China

## Abstract

We present here the draft genome sequence of *Lactobacillus brevis* strain 3M004, a probiotic that has potential for regulating quorum sensing. The strain was obtained from a type of aquafeed. The assembly consists of 2,459,326 bp and contains 33 contigs, with a G+C content of 45.10%.

## GENOME ANNOUNCEMENT

*Lactobacillus brevis* strain 3M004 was obtained from a type of aquafeed. Some strains of this species have been reported to act against influenza in elementary schoolchildren and have anti-inflammatory effects on periodontal disease (1, 2). This strain could degrade acyl-homoserine lactones (AHLs) as quorum-sensing (3) signaling molecules efficiently, and the strain does not produce AHLs, which indicates that it has potential for regulating quorum sensing.

A TIANamp bacterial DNA kit (DP302-02, TIANGEN Biotech Co, Ltd, Beijing, China) was used to extract genomic DNA from *L. brevis* strain 3M004. A paired-end library with an insert size of 350 bp was constructed and sequenced using the Illumina HiSeq platform with a PE150 strategy. Then, the high-quality reads, without PCR adapter sequences, were assembled with SOAPdenovo (4, 5) to generate scaffolds, and all reads were used for further gap closure. A total of 667 Mb of high-quality sequence data were obtained, and the resulting assembly consisted of 2,459,326 bp comprising 33 contigs (the longest was 435,481 bp; *N*_50_ was 299,821 bp) with a G+C content of 45.10%. With gene prediction, a total of 2,410 genes in the genome assembly of *L. brevis* strain 3M004 were found by GeneMarkS (6) with integrated and heuristic model parameters. Gene annotation was performed with BLAST (7) searches (E value less than 1e^−5^ and minimal alignment length larger than 40%) against four databases; as a result, 1,325, 1,704, 2,350, and 1,664 functional genes were annotated from KEGG (8), COG (9), NR, and GO (10), respectively.

### Accession number(s).

The genome sequence of *L. brevis* strain 3M004 was deposited at DDBJ/ENA/GenBank under the accession number NFZZ00000000. The version described in this paper is the first version, NFZZ01000000.
